# The Effects of Korean Red Ginseng on Biological Aging and Antioxidant Capacity in Postmenopausal Women: A Double-Blind Randomized Controlled Study

**DOI:** 10.3390/nu13093090

**Published:** 2021-09-02

**Authors:** Tae-Ha Chung, Ji-Hye Kim, So-Young Seol, Yon-Ji Kim, Yong-Jae Lee

**Affiliations:** 1 Department of Family Medicine, Wonju Severance Christian Hospital, Yonsei University Wonju College of Medicine, Wonju 26426, Korea; medeus115@yonsei.ac.kr; 2 Department of Medicine, Graduate School of Medicine, Yonsei University, Seoul 03722, Korea; 3 Department of Health Promotion, Severance Check-Up, Yonsei University Health System, Seoul 03722, Korea; bles4you@yuhs.ac (J.-H.K.); redyonji@gmail.com (Y.-J.K.); 4 Department of Internal Medicine, Gangnam Severance Hospital Biomedical Research Center, Yonsei University College of Medicine, Seoul 06273, Korea; syseol0916@yuhs.ac; 5 Department of Family Medicine, Gangnam Severance Hospital, Yonsei University College of Medicine, Seoul 06273, Korea

**Keywords:** Korean red ginseng, antioxidant activity, biological aging, fatigue, postmenopausal women

## Abstract

Postmenopausal women are vulnerable to aging and oxidative stress due to reduced estrogen. Previous studies have shown that Korean red ginseng (KRG) has beneficial effects on aging and antioxidant capacity. Therefore, we evaluated the effects of KRG on biological aging and antioxidant capacity in postmenopausal women. This study conducted a double-blinded, placebo-controlled clinical trial. The participants were randomly administered KRG or a placebo, and the following metrics were measured: mitochondria DNA (mtDNA) copy number as an indicator of biological aging and, total antioxidant status (TAS) as a marker of antioxidant capacity. Clinical symptoms of fatigue, as measured by the fatigue severity scale, were assessed before and after KRG administration. There were 63 participants, of whom 33 received KRG and 30 received a placebo. The mtDNA copy number (KRG group: 1.58 ± 2.05, placebo group: 0.28 ± 2.36, *p* = 0.023) and TAS (KRG group: 0.11 ± 0.25 mmol/L, placebo group: −0.04 ± 0.16 mmol/L, *p* = 0.011) increased and the fatigue severity scale (KRG group: −7 ± 12, placebo group: −1 ± 11, *p* = 0.033) decreased significantly more in the KRG group than the placebo group. KRG significantly increased the mtDNA copy number, total antioxidant status, and improved symptoms of fatigue in postmenopausal women.

## 1. Introduction

Ginseng was traditionally used in East Asian countries, including Korea, China, and Japan, as a functional health food and natural therapeutic medicine, but it is increasingly being recognized as a natural health food internationally [1,2]. Korean red ginseng (KRG), the technical name of which is *Panax ginseng* Meyer, is produced by steaming and drying fresh, unpeeled ginseng. KRG has anti-cancer and antioxidant properties and consuming it improves immune system activity, fatigue symptoms, blood circulation, and memory function [2].

Although no exact mechanism has been identified for the aging process in women after menopause, several epidemiological studies have shown that there are many diseases related to aging in postmenopausal women. In a prospective study of 12,134 Dutch women, age-adjusted mortality increased by 2% in women who began menopause more than a year earlier [3]. In a Korean study, Kim et al. analyzed data from the Korean National Health and Nutrition Examination Survey and found that postmenopausal women were 1.6 times more likely to develop a metabolic syndrome than premenopausal women [4]. A large cohort study on women, including postmenopausal women, showed that various metabolic parameters associated with type 2 diabetes and cardiovascular disease rates, both of which are closely linked to oxidative stress and chronic low-grade inflammation, dramatically increased after menopause began [5].

Mitochondrial dysfunction is associated with senescence [6]. Both mtDNA mutations and decreases in mtDNA copy number interrupt mitochondrial function, which may lead to aging [7,8]. In addition, free radicals accumulate in macromolecules, such as fat, DNA, and protein, and oxidative stress leads to cellular senescence, which induces the expression of various inflammatory cytokines and proteins, which in turn promote aging [9,10]. Previous clinical studies of the antioxidant effects of KRG showed that antioxidant indicators such as superoxide dismutase (SOD), glutathione peroxidase (GPx), and catalase increased in postmenopausal women [11]. However, the measurement of these antioxidant indicators has limitations in primary clinical settings. As the process of examination is complex and mainly laboratory-measurable, it cannot easily be used in clinical practice. Meanwhile, a colorimetry diagnosis technique has recently been developed for the assessment of antioxidative activity using simple blood tests in clinical practice. This method evaluates all antioxidative indicators, including both enzymatic and non-enzymatic molecules in the blood, and yields total antioxidant status (TAS) [12]. 

Unlike previous studies, in this study, the mtDNA copy number of lymphocytes was used to determine biological aging, and TAS was measured to evaluate antioxidant capability with diagnostic indicators that can easily be verified in the primary clinical setting. Furthermore, we used the Fatigue Severity Scale (FSS) questionnaire as an indicator of clinical symptoms for fatigue. Therefore, this study aimed to investigate the effect of KRG primarily on biological aging and antioxidant capacity, also to evaluate how it affects clinical fatigue symptoms.

## 2. Materials and Methods

### 2.1. Study Design and Participants

This study comprised an 8-week, randomized, double-blind, placebo-controlled, clinical trial. Participants were examined at the Severance Health Check-up, Severance Hospital, Yonsei University Health System, in Seoul, Korea, between August 2019 and March 2021. This study’s protocol was approved by the Institutional Review Board of Gangnam Severance Hospital (IRB no.: 3-2019-0089) and registered at the Clinical Research Information Service (CRIS, KCT0006506). This study was performed in compliance with the Declaration of Helsinki. 

Eligible participants were postmenopausal women aged 46–69 years who could communicate in Korean naturally and who were not participating in any other interventional studies. Additionally, participants had to have no physical restrictions on movement. Those who were excluded were current smokers; those who consumed more than 3 standard alcoholic drinks more than two days per week; who had uncontrolled hypertension, defined as systolic blood pressure (SBP) ≥ 180 mmHg or diastolic blood pressure (DBP) ≥ 100 mmHg; who had uncontrolled diabetes mellitus, defined as having fasting plasma glucose ≥ 200 mg/dL; who had been diagnosed with liver disease and aspartate aminotransferase (AST) or alanine aminotransferase (ALT) levels more than three times the upper limit; who had been diagnosed with kidney, cardiovascular, or cerebral vascular disease; who had any form of cancer or were undergoing cancer treatment; or who were taking other health supplements or female hormones. Written informed consent was obtained from all patients before participation.

**Figure 1:**
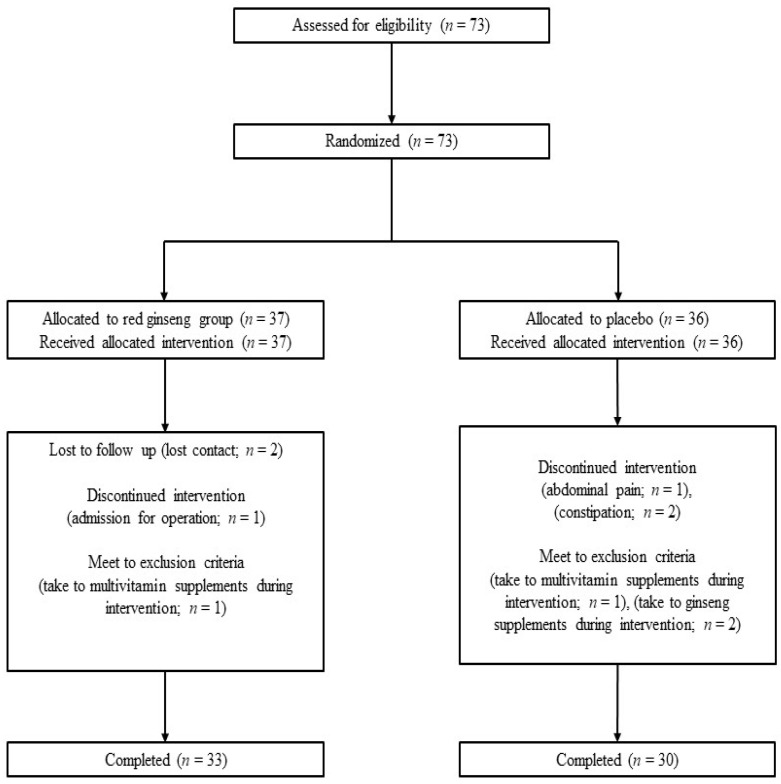
Flowchart of the study.

A total of 73 participants were enrolled in this study, 37 of whom were randomly placed in the KRG group and 36 of whom were randomly placed in the placebo group. During the study, 4 participants from the KRG group were excluded, 2 of whom stopped participating for unknown reasons, 1 of whom was hospitalized to receive surgery, and 1 of whom took multivitamin supplements. Six participants were excluded from the placebo group, 1 of whom had abdominal pain, 2 of whom had constipation, 1 of whom took multivitamin supplements, and 2 of whom took other red ginseng products. A total of 33 participants in the KRG group and 30 participants in the placebo group completed the study. Figure 1 shows a flow chart for how the study was conducted.

### 2.2. Randomization 

Participants were randomly assigned to take a 500 mg KRG or placebo tablet four times daily. Participants were assigned to each group in an even ratio by sequentially assigning them in alternating order by their assignment code. These codes were generated using SAS version 9.4 (SAS Institute, Inc., Cary, NC, USA). Both researchers and participants were blinded to the assignments throughout the study.

### 2.3. Korean Red Ginseng and Placebo Supplements

Participants in the KRG group received 2 g of KRG tablet per day. The tablets contained 8.03 mg/g of ginsenoside Rb1, 3.29 mg/g of Rc, 2.80 mg/g of Rb2, 2.50 mg/g of Rg3, 1.47 mg/g of Rf, 1.29 mg/g of Re, 1.18 mg/g of Rg1, and 1.0 mg/g of Rd. The KRG tablets were prepared by dehydrating KRG extract (3 g of KRG extract per 2 g tablet). The placebo tablets contained cornstarch and cellulose with the same flavor as the KRG tablets. The KRG and placebo tablet were provided by the study sponsor (The Korean Society of Ginseng, Seoul, Korea).

### 2.4. Data Collection

Each participant completed a questionnaire about lifestyle, medical history, and health supplements. Self-reported cigarette smoking, alcohol consumption, and physical activity were extracted from the questionnaires. Current alcohol consumption was defined as drinking less than 2 cups of alcoholic beverages a day per week. Participants were asked about their physical exercise on a weekly basis, and physical activity was defined as exercising more than 3 times per week with moderate-to-vigorous intensity. Body mass and height were measured to the nearest 0.1 kg and 0.1 cm, respectively, while participants wore light indoor clothing and no shoes. Body mass index was defined as the participant’s weight in kilograms divided by the square of their height in meters. SBP and DBP were measured using the patient’s right arm with a standard mercury sphygmomanometer (Kensei Industry Co., Ltd., Takasai Shimotsuma, Japan). Participants were defined as having hypertension if their SBP ≥ 140 mmHg, their DBP ≥ 90 mmHg, they were taking anti-hypertension medication, or they had been diagnosed with hypertension by a physician. Participants were defined as having diabetes if their fasting plasma glucose ≥ 126 mg/dL, their glycated hemoglobin level ≥ 6.5%, they were taking anti-diabetes medication, or they had been diagnosed with diabetes by a physician. Participants were defined as having dyslipidemia if their total cholesterol ≥ 240 mg/dL, low-density lipoprotein (LDL) cholesterol ≥ 160 mg/dL, triglycerides ≥ 150 mg/dL, or high-density lipoprotein (HDL) cholesterol < 50 mg/dL, they were taking anti-dyslipidemia medication, or they had been diagnosed with dyslipidemia by a physician. Participants were defined as being postmenopausal if they had not had a menstrual period for at least the preceding 12 months in the absence of a clear biological or physiological cause. 

Blood samples were obtained after 8 h of overnight fasting. Leukocyte counts were measured using an ADVIA 2120i Hematology System (Siemens Healthcare Diagnostic, Inc., Tarrytown, NY, USA). Fasting plasma glucose, total cholesterol, triglycerides, LDL cholesterol, HDL cholesterol, AST, ALT, and gamma-glutamyltransferase (gamma-GT) were measured with an ADVIA 1800 Clinical Chemistry System (Siemens Healthcare Diagnostic, Inc., Tarrytown, NY, USA).

### 2.5. Dependent Variable Assessment 

#### 2.5.1. Total Antioxidant Status 

TAS was measured using a TBA c8000 automated colorimeter (Toshiba Medical system Ltd., Tokyo, Japan). The antioxidative molecules in the sample reduce dark blue-green-colored 2,2′-azinobis(3-ethylbenzo-thiazoline-6-sulfonate) radicals into a colorless form according to antioxidant concentrations and capacities. The change in absorbance at 660 nm is related to the total amount of antioxidants in the sample. The TAS assay was calibrated with Trolox equivalent, a stable, standard antioxidant solution and a vitamin E analog [12]. 

#### 2.5.2. Mitochondria DNA Copy Number

All DNA samples were extracted from ethylenediaminetetraacetic acid (EDTA)-treated blood. High-quality genomic DNA was extracted from participants’ peripheral blood using a QIAamp DNA Mini Kit (Qiagen, Valencia, CA, USA) according to the manufacturer’s protocol. The concentration and purity of total DNA were controlled by taking absorbance readings at 260 and 280 nm using a Nanodrop 1000 spectrophotometer (preQLab Biotechnology, Erlangen, Germany). The relative mtDNA copy number was measured by quantitative real-time PCR. Two pairs of primers were designed and used to quantify the mtDNA copy number. The first primer pair was used to amplify the ND5 gene in the mtDNA.

The forward primer (ND5-F) was 5′-TTCATCCCTGTAGCATTGTTCG-3′ and the reverse primer (ND5-R) was 5′-GTTGGAATAGGTTGTTAGCGGTA-3′. The second primer pair was used to amplify the nuclear DNA rRNA 18S. The forward primer (18S-F) was 5′- GAGAAACGGCTACCACATCC-3′ and the reverse primer (18S-R) was 5′-GCCTCGAAAGAGTCCTGTATTG-3′. Ten uL of the PCR reaction mixture for mtDNA amplification consisted of 2 X SYBR Green Mastermix (Applied Biosystems, Waltham, MA, USA), 100 pmol/uL of ND5-F or 18S-F primer, 100 pmol/uL of ND5-R or 18S-R primer, and 10 ng of genomic DNA. The samples were heated to 95 °C for 10 min followed by 40 cycles of heating to 95 °C for 15 s and then cooling to 55 °C for 1 min. Each sample was run in triplicate in a 96-well plate with an Applied Biosystems 7300 real-time PCR system (Applied Biosystems, Waltham, MA, USA). The relative copy number was calculated from the threshold cycle value (ΔCt), which was defined as Ct18S–CtND5, where the mean amount of mtDNA = 2^−ΔCt^. The fold change of the mtDNA copy number was calculated as the amount of ND5, the target gene, DNA normalized to the amount of 18S, the reference gene, DNA in a treated sample.

#### 2.5.3. Fatigue Severity Scale 

The FSS is a popular tool for measuring fatigue symptoms [13]. It has been validated for various diseases, such as multiple sclerosis, systemic lupus erythematosis, Parkinson’s disease, and stroke [14,15,16]. It consists of 9 items, each of which can receive a score of 1–7 points. Fatigue severity is the sum of each item’s score for a total score range of 7–63 where higher scores indicate more severe fatigue symptoms. 

### 2.6. Statistical Analyses

We analyzed the data based on the law of large numbers and the central limit theorem because each group had at least 30 participants. The groups’ baseline characteristics were compared using independent *t*-tests for continuous variables and chi-squared tests for categorical values. We used a paired *t*-test to determine whether the groups’ metabolic parameters differed after the intervention. The mean differences in changes between the groups were compared using independent two-sample *t*-tests. All statistical analyses were performed using SPSS version 25.0 for Windows (IBM, Armonk, NY, USA). All statistical tests were two-sided and *p*-values < 0.05 were considered statistically significant.

## 3. Results

The groups’ baseline characteristics are presented in Table 1. The groups had similar anthropometric parameters. The average age of the KRG group was 58.7 ± 4.2 years old, and that of the placebo group was 59.7 ± 4.2 years old. There were no significant differences between the two groups’ results in terms of SBP, DBP, waist circumference, body mass index, fasting plasma glucose levels, triglyceride, HDL cholesterol, LDL cholesterol, AST, ALT, gamma-GT level, mtDNA copy number, TAS, FSS, alcohol consumption rates, physical activity rate, or prevalence of hypertension, diabetes, or dyslipidemia. However, the KRG group’s average leukocyte count was 4890 ± 931 cells/μL, while that of the placebo group was 5553 ± 1361 cells/μL (*p* = 0.027), and the KRG group’s total cholesterol level was 230 ± 31 mg/dL, while that of the placebo group was 210 ± 41 mg/dL (*p* = 0.034). 

Table 2 presents the changes in the metabolic parameters in the two groups over the course of the study. Body mass index, SBP, DBP, and hematologic parameters did not change significantly in either group. The KRG group’s LDL cholesterol tended to decrease from 136 ± 38 to 127 ± 34 mg/dL compared to the placebo group (*p*=0.067). However, the two groups had no significant difference in mean changes of LDL cholesterol (*p* = 0.064).

Table 3 presents data regarding this study’s primary variables of interest, including antioxidant capacity and biological aging indicators. TAS increased significantly in the KRG group, from 1.42 ± 0.16 to 1.53 ± 0.19 mmol/L (*p* = 0.021), but did not statistically significantly change in the placebo group, decreasing from 1.44 ± 0.14 to 1.40 ± 0.17 mmol/L (*p* = 0.258). The KRG group’s mean change in TAS of 0.11 ± 0.25 mmol/L was significantly greater than that of the placebo group at −0.04 ± 0.16 mmol/L (*p* = 0.011).

The biological indicator, mtDNA copy number was significantly higher in the KRG group, increasing from 4.36 ± 2.54 to 5.93 ± 3.33 (*p* < 0.001), but not in the placebo group, increasing from 4.70 ± 3.94 to 4.98 ± 3.17 (*p* = 0.520). The mean change in mtDNA copy number was significantly different between the groups, with the KRG group’s change being 1.58 ± 2.05 and the placebo group’s being 0.28 ± 2.36 (*p* = 0.023). Furthermore, the fold change refers to the multiple when the baseline mtDNA copy number is considered to be 1. The mtDNA copy number fold change in the KRG group was 1.44 ± 0.46, which was significantly higher than that of the placebo group of 1.15 ± 0.40 (*p* = 0.012).

**Figure 2:**
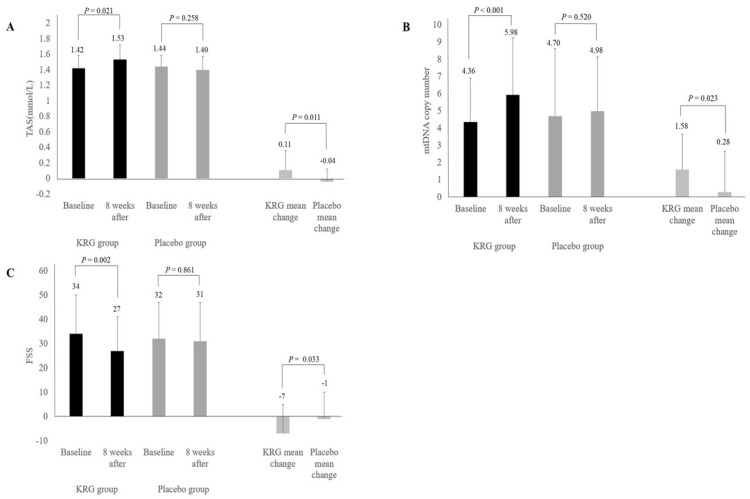
Changes in major results of the KRG and placebo groups before and after 8 weeks (bars mean standard deviation): (**A**) Total antioxidant status; (**B**) mtDNA copy number; (**C**) Fatigue severity scale. KRG, Korean red ginseng.

In addition, FSS scores decreased significantly in the KRG group from 34 ± 16 to 27 ± 14 (*p* = 0.002) but did not significantly change in the placebo group, decreasing from 32 ± 15 to 31 ± 16 (*p* = 0.861). The mean change in the KRG group’s FSS scores was −7 ± 12, which was significantly different from that of the placebo group of −1 ± 11 (*p* = 0.033) (Table 4). The significant results of this study are summarized in Figure 2.

## 4. Discussion

This study comprised a randomized, double-blind, placebo-controlled clinical trial to evaluate the effect of KRG consumption on biological aging, antioxidant capacity, and fatigue symptoms in postmenopausal women. We found that taking 2 g/day of KRG for 8 weeks increased mtDNA copy number and TAS and reduced fatigue symptoms more than the placebo. Our findings are consistent with those of previous studies on the effectiveness of KRG. 

First, in this study, KRG was shown to increase the mtDNA copy number more than the placebo. A previous study showed that KRG improved exercise performance, increased the ATP production capacity of muscle cells, and stimulated myoblast differentiation in mice [17]. An animal study showed that the mtDNA copy number and messenger RNA expression levels of mitochondrial biogenesis-related transcription factors increased more in those with diabetes who took KRG than those who took a placebo [18]. A clinical trial showed that the mtDNA copy number for men with metabolic syndromes increased when they consumed 3 g/day of KRG and the mean change in their mtDNA copy number was significantly higher than that of the group who consumed a placebo [19]. 

The following mechanism can be considered. Peroxisome proliferator-activated receptor-gamma co-activator 1α (PGC-1α) plays a pivotal role in the regulation of mitochondrial biogenesis. It activates nuclear respiratory factor (NRF)-1 and NRF-2, thereby promoting the expression of mitochondrial transcription factor A (TFAM), which upregulates mitochondrial DNA transcription and replication [20,21]. Moreover, Kim et al. reported that the Rg3 component in KRG increased the expression of PGC-1α, NRF-1, and TFAM in an in vitro model [22]. Additionally, Shin et al. showed that KRG improved swimming performance in mice and enhanced TFAM and NRF-1 gene expression levels [17]. Therefore, KRG may lead to improvements in the PGC-1α-NRF1-TFAM pathway, which contributes to the increased mtDNA copy number. 

The mtDNA copy number is associated with aging and oxidative stress [6,23]. Therefore, our result suggests that consuming KRG can increase mitochondrial functioning, which is negatively associated with biological aging. 

Second, the KRG group had significantly higher TAS levels following the intervention than the placebo group did. Studies on the antioxidant activity of KRG in animal models have shown that the levels of antioxidant indicators, such as SOD, GPx, and catalase, increased and the levels of the oxidative stress marker MDA decreased [24,25,26]. Seo et al. showed that postmenopausal women who took 3 g/day of KRG for 12 weeks had significantly higher SOD levels than those who took a placebo [11]. Another clinical trial in Korea showed that healthy adults who took 6 g/day of KRG had greater increases in GPx, SOD, and catalase activity than those who took a placebo [27]. Our results are consistent with these studies. However, a significant difference between this study and the mentioned studies is that antioxidant indicators, which can easily be detected in primary care settings, were used in this study rather than markers such as SOD, GPx, and catalase that are much harder to detect in clinical settings. And, even if the dose and treatment duration of KRG were lower than in previous studies, it had an effect on antioxidant capacity. Although the antioxidant mechanism of KRG is not clearly understood, the following mechanism can be considered. First of all, it may improve antioxidant capacity by downregulating reactive oxygen species (ROS)-stimulated mitogen-activated protein kinase and protein kinase B pathways that are involved in cell survival and growth [28,29]. Furthermore, KRG can upregulate nuclear factor erythroid 2-related factor 2 (Nrf 2)-mediated expression of heme oxygenase 1 as a ROS scavenging factor [30]. Additionally, the intracellular concentration of glutathione (GSH) was increased and attenuated the reduction of GSH in the cell levels [31].

Third, the KRG group showed improvements in fatigue symptoms. A meta-analysis reported that red ginseng intake does not statistically significantly reduce fatigue symptoms [32]. Another randomized, placebo-controlled study did not show that KRG consumption resulted in a significant decrease in fatigue symptoms for the full sample, but a subgroup analysis showed that it did for people over 50 years old with moderate fatigue symptoms [33]. In this study, the KRG could reduce fatigue symptoms in postmenopausal women as confirmed with statical significance after the intervention. 

On the other hand, the mean LDL cholesterol levels in the KRG group decreased from 136 to 127 mg/dL, but they did not decrease in the placebo group. However, this decrease was not statistically significant (*p* = 0.067). A previous study showed that administering 6 g/day of *P. ginseng* extract for 8 weeks decreased LDL cholesterol in males [34]. Another clinical study with hyperlipidemic patients reported that LDL cholesterol decreased significantly in the red ginseng (2 g/day) and placebo group, but there was no significant difference between the two groups [35]. The subjects of that study were young, male, and hyperlipidemic, while this study was conducted on postmenopausal women. Therefore, this difference seems to indicate a dissimilarity in the study results. However, even though the baseline LDL cholesterol level was in the normal range, it showed a tendency to decrease after the intervention in postmenopausal women. A large sample and long-term clinical study are necessary to evaluate the effect of KRG on lipid metabolism in the future.

This study has several strengths. Most related studies used SOD, GPx, and MDA as indicators of antioxidant capacity, but these markers are difficult to detect in general clinical settings. We confirmed that the total antioxidant ability was increased in the KRG group by quantitatively measuring various factors involved in antioxidant capacity in each individual’s blood sample, differently from the previous methods. In addition, this study showed that the mtDNA copy number, which is related to mitochondrial function, energy metabolism, and the aging process, was enhanced, indicating that KRG might protect against aging and increase cellular metabolism. In addition, we objectively evaluated fatigue symptoms through validated questionnaires and showed that the KRG group’s fatigue symptoms decreased. Finally, estrogen directly protects against mitochondrial apoptosis and reduces oxidative stress [36]. Thus, this study was conducted with postmenopausal women whose biological aging would be faster and whose antioxidant activity would be lower than that of premenopausal women because they have low estrogen. Furthermore, postmenopausal women were selected as the study subjects to control the effects of lifestyle habits such as smoking and drinking, which are relatively more linked to aging and oxidative stress in women than in men. Therefore, it is significant that the study was conducted on postmenopausal women with decreased estrogen levels, which is related to the aging process and antioxidant capacity.

This study is the first randomized, double-blind, placebo-controlled study to investigate the comprehensive efficacy of KRG administration on biological aging, antioxidant activity, and clinical symptoms in Korean postmenopausal women. However, this study also has some limitations. The first limitation is that the KRG group was administered 2 g/day of KRG for 8 weeks. Previous clinical studies that evaluated changes in mtDNA copy number, antioxidant indicators, and clinical symptoms varied the dose of KRG from 1 to 6 g/day and the duration of administration from 2 to 12 weeks. These ranges indicate that the dose and duration of KRG administration are not strongly agreed upon, so use of higher doses or longer administration durations in this study may have produced different outcomes, including LDL cholesterol. However, a daily dose of 2 g of KRG is generally available as a healthy functional supplement in the market. Accordingly, in this study, we selected 2 g per day, the dosage for safety KRG intake as a general health functional food. Therefore, determining the optimal dose and duration for KRG intervention should be further investigated in the future. The second limitation is that, although we told the participants to keep their usual lifestyle during the clinical trial, we could not strictly control each individual’s behaviors, which could affect biological aging, antioxidant capacity, and clinical symptoms. The third limitation is that the participants were all Korean postmenopausal women, so the results may not be broadly generalizable to other populations, such as men, premenopausal women, and different ethnicities.

## 5. Conclusions

This study examined the effects of KRG consumption on the primary outcomes for variables of interest, which were changes in biological aging marker and antioxidant capacity, and secondary variables of interest, which were fatigue symptoms, in Korean postmenopausal women. The results showed that administering 2 g/day of KRG for 8 weeks increased the mtDNA copy number, increased antioxidant activity, and reduced fatigue symptoms more than the placebo.

## Figures and Tables

**Table 1:** Baseline characteristics of study participants.

	KRG	Placebo	*p*-Value
*N*	33	30	
Age, years	58.7 ± 4.2	59.7 ± 4.2	0.330
SBP (mmHg)	120.7 ± 15.3	125.0 ± 11.1	0.207
DBP (mmHg)	73.0 ± 11.6	77.2 ± 7.9	0.104
Waist circumference (cm)	79.3 ± 6.9	80.9 ± 7.2	0.371
BMI (kg/m^2^)	23.7 ± 2.4	23.8 ± 3.1	0.844
Luekocyte count (cells/μL)	4890 ± 931	5553 ± 1361	0.027
Fasting plasma glucose (mg/dL)	95 ± 11	96 ± 17	0.831
Total cholesterol (mg/dL)	230 ± 31	210 ± 41	0.034
Triglyceride (mg/dL)	129 ± 66	116 ± 52	0.392
HDL cholesterol (mg/dL)	68 ± 16	65 ± 16	0.373
LDL cholesterol (mg/dL)	136 ± 38	124 ± 36	0.205
AST (U/L)	27 ± 8	25 ± 4	0.360
ALT (U/L)	23 ± 9	23 ± 7	0.873
Gamma-GT (U/L)	17 ± 11	16 ± 7	0.606
Alcohol consumption, *n* (%)	3 (9.1)	5 (16.7)	0.367
Physical activity, *n* (%)	17 (51.5)	10 (33.3)	0.145
HTN, *n* (%)	4 (12.1)	4 (13.3)	0.885
Diabetes, *n* (%)	1 (3.0)	2 (6.7)	0.498
Dyslipidemia, *n* (%)	5 (15.2)	7 (23.3)	0.409
TAS (mmol/L)	1.42 ± 0.16	1.44 ± 0.14	0.690
mtDNA copy number	4.35 ± 2.54	4.70 ± 3.94	0.678
FSS	34 ± 16	32 ± 15	0.619

*p*-values were calculated using the independent two-sample. *t*-test for continuous values and the chi-square test for categorical value. KRG, Korean red ginseng; BMI, body mass index; SBP, systolic blood pressure; DBP, diastolic blood pressure; AST, aspartate aminotransferase; ALT, alanine aminotransferase; Gamma-GT, gamma-glutamyltransferase; HTN, hypertension; TAS, total anti-oxidative status; mtDNA copy number, mitochondria DNA copy number; FSS, fatigue severity scale.

**Table 2:** Changes in metabolic parameters of the KRG and placebo groups before and after 8 weeks.

	KRG			Placebo			
	Baseline	8 Weeks	*p*-Value	Baseline	8 Weeks	*p*-Value	Changed *p*-Value
BMI (kg/m^2^)	23.7 ± 2.4	23.7 ± 2.40	0.755	23.8 ± 3.1	23.9 ± 3.00	0.811	
Change	0 ± 0.5			0.1 ± 0.1			0.972
SBP (mmHg)	120.7 ± 15.3	120.3 ± 14.9	0.822	125.0 ± 11.1	124.5 ± 12.7	0.819	
Change	−0.4 ± 9.9			−0.5 ± 11			0.973
DBP (mmHg)	73.3 ± 11.6	74.4 ± 9.3	0.255	77.2 ± 9.6	77.3 ± 7.9	0.948	
Change	1.4 ± 6.7			0.1 ± 8			0.511
Luekocyte count (cells/μL)	4890 ± 931	4886 ± 1385	0.800	5553 ± 1361	5354 ± 1380	0.414	
Change	−4 ± 970			−199 ± 1317			0.505
Fasting plasma glucose (mg/dL)	95 ± 11	95 ± 8	0.969	96 ± 17	96 ± 16	0.736	
Change	0 ± 9			0 ± 8			0.847
Total cholesterol (mg/dL)	230 ± 31	223 ± 31	0.172	210 ± 41	209 ± 44	0.773	
Change	−7 ± 30			−1 ± 31			0.465
Triglyceride (mg/dL)	129 ± 66	137 ± 124	0.560	116 ± 52	107 ± 44	0.258	
Change	8 ± 80			−9 ± 41			0.305
HDLcholesterol (mg/dL)	68 ± 16	67 ± 16	0.549	65 ± 16	63 ± 15	0.240	
Change	−1 ± 7			−2 ± 8			0.619
LDL cholesterol (mg/dL)	136 ± 38	127 ± 34	0.067	124 ± 36	128 ± 42	0.414	
Change	−9 ± 24			4 ± 26			0.064
AST (U/L)	27 ± 8	25 ± 4	0.334	25 ± 4	27 ± 6	0.103	
Change	−2 ± 8			2 ± 6			0.082
ALT (U/L)	23 ± 9	22 ± 9	0.608	23 ± 7	24 ± 9	0.536	
Change	−1 ± 7			1 ± 10			0.414
Gamma-GT (U/L)	17 ± 11	19 ± 12	0.395	16 ± 7	16 ± 5	0.346	
Change	−2 ± 7			0 ± 4			0.243

*p*-values were calculated using the paired *t*-test (difference within groups after intervention). Changed *p*-values were calculated using the independent two-sample *t*-test (mean change difference between two groups). KRG, Korean red ginseng; BMI, body mass index; SBP, systolic blood pressure; DBP, diastolic blood pressure; AST, aspartate aminotransferase; ALT, alanine aminotransferase; Gamma-GT, gamma-glutamyltransferase.

**Table 3:** Changes in antioxidant capacity and biological aging parameter of the KRG and placebo groups before and after 8 weeks.

	KRG			Placebo			
	Baseline	8 Weeks	*p*-Value	Baseline	8 Weeks	*p*-Value	Changed *p*-Value
TAS (mmol/L)	1.42 ± 0.16	1.53 ± 0.19	0.021	1.44 ± 0.14	1.40 ± 0.17	0.258	
Change	0.11 ± 0.25			−0.04 ± 0.16			0.011
mtDNA copy number	4.36 ± 2.54	5.93 ± 3.33	<0.001	4.70 ± 3.94	4.98 ± 3.17	0.520	
Change	1.58 ± 2.05			0.28 ± 2.36			0.023
Fold change	1.44 ± 0.46			1.15 ± 0.40			0.012

*p*-values were calculated using the paired *t*-test (difference within groups after intervention). Changed *p*-values were calculated using the independent two-sample *t*-test (mean change difference between two groups). KRG, Korean red ginseng; TAS, total anti-oxidative status; mtDNA copy number, mitochondria DNA copy number.

**Table 4:** Changes in questionnaire scores (FSS) of the KRG and placebo groups before and after 8 weeks.

	KRG			Placebo			
	Baseline	8 Weeks	*p*-Value	Baseline	8 Weeks	*p*-Value	Changed *p*-Value
FSS	34 ± 16	27 ± 14	0.002	32 ± 15	31 ± 16	0.861	
Change	−7 ± 12			−1 ± 11			0.033

*p*-values were calculated using the paired *t*-test (difference within groups after intervention). Changed *p*-values were calculated using the independent two-sample *t*-test (mean change difference between two groups). KRG, Korean red ginseng; FSS, fatigue severity scale.

## Data Availability

Not applicable.

## References

[1] Baeg I.H., So S.H. (2013). The world ginseng market and the ginseng (Korea). J. Ginseng Res..

[2] So S.H., Lee J.W., Kim Y.S., Hyun S.H., Han C.K. (2018). Red ginseng monograph. J. Ginseng Res..

[3] Ossewaarde M.E., Bots M.L., Verbeek A.L., Peeters P.H., van der Graaf Y., Grobbee D.E., van der Schouw Y.T. (2005). Age at menopause, cause-specific mortality and total life expectancy. Epidemiology.

[4] Kim H.M., Park J., Ryu S.Y., Kim J. (2007). The effect of menopause on the metabolic syndrome among Korean women: The Korean national health and nutrition examination survey, 2001. Diabetes Care.

[5] Auro K., Joensuu A., Fischer K., Kettunen J., Salo P., Mattsson H., Niironen M., Kaprio J., Eriksson J.G., Lehtimaki T. (2014). A metabolic view on menopause and ageing. Nat. Commun..

[6] Balaban R.S., Nemoto S., Finkel T. (2005). Mitochondria, oxidants, and aging. Cell.

[7] Zhang R., Wang Y., Ye K., Picard M., Gu Z. (2017). Independent impacts of aging on mitochondrial DNA quantity and quality in humans. BMC Genom..

[8] Lopez-Otin C., Blasco M.A., Partridge L., Serrano M., Kroemer G. (2013). The hallmarks of aging. Cell.

[9] Liguori I., Russo G., Curcio F., Bulli G., Aran L., Della-Morte D., Gargiulo G., Testa G., Cacciatore F., Bonaduce D. (2018). Oxidative stress, aging, and diseases. Clin. Interv. Aging.

[10] Pandey K.B., Rizvi S.I. (2010). Markers of oxidative stress in erythrocytes and plasma during aging in humans. Oxid. Med. Cell. Longev..

[11] Seo S.K., Hong Y., Yun B.H., Chon S.J., Jung Y.S., Park J.H., Cho S., Choi Y.S., Lee B.S. (2014). Antioxidative effects of Korean red ginseng in postmenopausal women: A double-blind randomized controlled trial. J. Ethnopharmacol..

[12] Erel O. (2004). A novel automated direct measurement method for total antioxidant capacity using a new generation, more stable abts radical cation. Clin. Biochem..

[13] Krupp L.B., LaRocca N.G., Muir-Nash J., Steinberg A.D. (1989). The fatigue severity scale. Application to patients with multiple sclerosis and systemic lupus erythematosus. Arch. Neurol..

[14] Mills R., Young C., Nicholas R., Pallant J., Tennant A. (2009). Rasch analysis of the fatigue severity scale in multiple sclerosis. Mult. Scler..

[15] Mattsson M., Moller B., Lundberg I., Gard G., Bostrom C. (2008). Reliability and validity of the fatigue severity scale in swedish for patients with systemic lupus erythematosus. Scand. J. Rheumatol..

[16] Herlofson K., Larsen J.P. (2002). Measuring fatigue in patients with parkinson’s disease—The fatigue severity scale. Eur. J. Neurol..

[17] Shin E.J., Jo S., Choi S., Cho C.W., Lim W.C., Hong H.D., Lim T.G., Jang Y.J., Jang M., Byun S. (2020). Red ginseng improves exercise endurance by promoting mitochondrial biogenesis and myoblast differentiation. Molecules.

[18] Park J.K., Shim J.Y., Cho A.R., Cho M.R., Lee Y.J. (2018). Korean red ginseng protects against mitochondrial damage and intracellular inflammation in an animal model of type 2 diabetes mellitus. J. Med. Food.

[19] Jung D.H., Lee Y.J., Kim C.B., Kim J.Y., Shin S.H., Park J.K. (2016). Effects of ginseng on peripheral blood mitochondrial DNA copy number and hormones in men with metabolic syndrome: A randomized clinical and pilot study. Complement. Ther. Med..

[20] Taherzadeh-Fard E., Saft C., Akkad D.A., Wieczorek S., Haghikia A., Chan A., Epplen J.T., Arning L. (2011). Pgc-1alpha downstream transcription factors nrf-1 and tfam are genetic modifiers of huntington disease. Mol. Neurodegener..

[21] Gureev A.P., Shaforostova E.A., Popov V.N. (2019). Regulation of mitochondrial biogenesis as a way for active longevity: Interaction between the nrf2 and pgc-1alpha signaling pathways. Front. Genet..

[22] Kim M.J., Koo Y.D., Kim M., Lim S., Park Y.J., Chung S.S., Jang H.C., Park K.S. (2016). Rg3 improves mitochondrial function and the expression of key genes involved in mitochondrial biogenesis in c2c12 myotubes. Diabetes Metab. J..

[23] Wallace D.C. (2005). A mitochondrial paradigm of metabolic and degenerative diseases, aging, and cancer: A dawn for evolutionary medicine. Annu. Rev. Genet..

[24] Kim Y.S., Kim Y.H., Noh J.R., Cho E.S., Park J.H., Son H.Y. (2011). Protective effect of Korean red ginseng against aflatoxin b1-induced hepatotoxicity in rat. J. Ginseng Res..

[25] Ramesh T., Kim S.W., Hwang S.Y., Sohn S.H., Yoo S.K., Kim S.K. (2012). Panax ginseng reduces oxidative stress and restores antioxidant capacity in aged rats. Nutr. Res..

[26] Yu S.Y., Yoon B.R., Lee Y.J., Lee J.S., Hong H.D., Lee Y.C., Kim Y.C., Cho C.W., Kim K.T., Lee O.H. (2014). Inhibitory effect of high temperature- and high pressure-treated red ginseng on exercise-induced oxidative stress in icr mouse. Nutrients.

[27] Kim J.Y., Park J.Y., Kang H.J., Kim O.Y., Lee J.H. (2012). Beneficial effects of Korean red ginseng on lymphocyte DNA damage, antioxidant enzyme activity, and ldl oxidation in healthy participants: A randomized, double-blind, placebo-controlled trial. Nutr. J..

[28] Bak M.J., Jun M., Jeong W.S. (2012). Antioxidant and hepatoprotective effects of the red ginseng essential oil in h(2)o(2)-treated hepg2 cells and ccl(4)-treated mice. Int. J. Mol. Sci..

[29] Park H.M., Kim S.J., Mun A.R., Go H.K., Kim G.B., Kim S.Z., Jang S.I., Lee S.J., Kim J.S., Kang H.S. (2012). Korean red ginseng and its primary ginsenosides inhibit ethanol-induced oxidative injury by suppression of the mapk pathway in tib-73 cells. J. Ethnopharmacol..

[30] Park S.H., Jang J.H., Chen C.Y., Na H.K., Surh Y.J. (2010). A formulated red ginseng extract rescues pc12 cells from pcb-induced oxidative cell death through nrf2-mediated upregulation of heme oxygenase-1 and glutamate cysteine ligase. Toxicology.

[31] Dong G.Z., Jang E.J., Kang S.H., Cho I.J., Park S.D., Kim S.C., Kim Y.W. (2013). Red ginseng abrogates oxidative stress via mitochondria protection mediated by lkb1-ampk pathway. BMC Complement. Altern. Med..

[32] Bach H.V., Kim J., Myung S.K., Cho Y.A. (2016). Efficacy of ginseng supplements on fatigue and physical performance: A meta-analysis. J. Korean Med. Sci..

[33] Sung W.S., Kang H.R., Jung C.Y., Park S.S., Lee S.H., Kim E.J. (2020). Efficacy of Korean red ginseng (panax ginseng) for middle-aged and moderate level of chronic fatigue patients: A randomized, double-blind, placebo-controlled trial. Complement. Ther. Med..

[34] Kim S.H., Park K.S. (2003). Effects of panax ginseng extract on lipid metabolism in humans. Pharmacol. Res..

[35] Delui M.H., Fatehi H., Manavifar M., Amini M., Ghayour-Mobarhan M., Zahedi M., Ferns G. (2013). The effects of panax ginseng on lipid profile, pro-oxidant: Antioxidant status and high-sensitivity c reactive protein levels in hyperlipidemic patients in iran. Int. J. Prev. Med..

[36] Borras C., Gambini J., Lopez-Grueso R., Pallardo F.V., Vina J. (2010). Direct antioxidant and protective effect of estradiol on isolated mitochondria. Biochim. Biophys. Acta.

